# A Fungal-Prokaryotic Consortium at the Basalt-Zeolite Interface in Subseafloor Igneous Crust

**DOI:** 10.1371/journal.pone.0140106

**Published:** 2015-10-21

**Authors:** Magnus Ivarsson, Stefan Bengtson, Henrik Skogby, Peter Lazor, Curt Broman, Veneta Belivanova, Federica Marone

**Affiliations:** 1 Department of Palaeobiology and Nordic Center for Earth Evolution, Swedish Museum of Natural History, Box 50007, SE-104 05 Stockholm, Sweden; 2 Department of Geosciences, Swedish Museum of Natural History, Box 50007, SE-104 05 Stockholm, Sweden; 3 Department of Earth Sciences, Uppsala University, Villavägen 16, SE-752 36 Uppsala, Sweden; 4 Department of Geological Sciences, Stockholm University, Svante Arrheniusväg 8, SE-106 91, Stockholm, Sweden; 5 Department of Palaeobiology, Swedish Museum of Natural History, Box 50007, SE-104 05 Stockholm, Sweden; 6 Swiss Light Source, Paul Scherrer Institute, CH-5232 Villigen, Switzerland; The University of Wisconsin - Madison, UNITED STATES

## Abstract

We have after half a century of coordinated scientific drilling gained insight into Earth´s largest microbial habitat, the subseafloor igneous crust, but still lack substantial understanding regarding its abundance, diversity and ecology. Here we describe a fossilized microbial consortium of prokaryotes and fungi at the basalt-zeolite interface of fractured subseafloor basalts from a depth of 240 m below seafloor (mbsf). The microbial consortium and its relationship with the surrounding physical environment are revealed by synchrotron-based X-ray tomographic microscopy (SRXTM), environmental scanning electron microscopy (ESEM), and Raman spectroscopy. The base of the consortium is represented by microstromatolites—remains of bacterial communities that oxidized reduced iron directly from the basalt. The microstromatolites and the surrounding basalt were overlaid by fungal cells and hyphae. The consortium was overgrown by hydrothermally formed zeolites but remained alive and active during this event. After its formation, fungal hyphae bored in the zeolite, producing millimetre-long tunnels through the mineral substrate. The dissolution could either serve to extract metals like Ca, Na and K essential for fungal growth and metabolism, or be a response to environmental stress owing to the mineral overgrowth. Our results show how microbial life may be maintained in a nutrient-poor and extreme environment by close ecological interplay and reveal an effective strategy for nutrient extraction from minerals. The prokaryotic portion of the consortium served as a carbon source for the eukaryotic portion. Such an approach may be a prerequisite for prokaryotic-eukaryotic colonisation of, and persistence in, subseafloor igneous crust.

## Introduction

The igneous portion of the oceanic crust has been recognized as a substantial microbial habitat and a major scientific frontier within geomicrobiology [1]. The total rock volume of the igneous oceanic crust is estimated to 2.3 X 10^18^ m^3^, which is 6–10 times that of the total marine sediments [2]. The upper 500–1000 meters of basaltic seafloor is characterized by average porosities of ~10%, which varies depending on the physical properties and age of the rock [2]. Roughly 60% of the igneous oceanic crust is hydrologically active and the total fluid volume corresponds to 2% of the total ocean [2]; thus the igneous oceanic crust is the largest aquifer system on Earth [3]. Indirectly, this means that the igneous ocean crust is the largest potential microbial habitat on Earth [2]. Considering its vastness and interconnectedness by pore space, a diversity of biotopes should be expected. However, its inaccessibility and the challenges with respect to sample contamination make the deep biosphere poorly understood.

In recent years a few molecular studies have been performed on seafloor-exposed basalts indicating a diverse biotope consisting of *Archaea*, *Bacteria* and even fungi [4–7]. In subseafloor basalts molecular studies are even more rare as they are difficult to access and sample without contamination. The presence of both *Archaea* [8] and *Bacteria* [9] has been suggested in subseafloor basalts, however, as well as microbial methane-cycling and sulfate-reduction [10]. In addition, paleontological material has been used to understand the deep communities. Granular and tubular alteration textures, ichnofossils, in volcanic glass were the first biostructures to be found and are still the most extensively studied fossils in subseafloor basalts [11, 12, 13], but filamentous fossilized microorganisms have also been found in open fractures or healed veins in basalts [14,15]. Unexpectedly, a majority of the filamentous fossils seem to be remains of fungi rather than filamentous prokaryotes. This is further corroborated by the presence of active fungal communities in marine sediments [16]. For a nutrient-limited environment such as the subseafloor basalts the presence of fungi has raised questions about their metabolic pathways [17] and access to energy and nutrient sources. Bengtson et al. [18] reported a microbial consortium consisting of fungi and two types of chemoautotrophs that seemingly existed in a symbiotic relationship. In such an arrangement, the fungi could scavenge the chemoautotrophic portion of the consortium, which fix dissolved carbon from fluids by oxidation of iron.

Because of the difficulty with which one can retrieve and investigate in situ microbial community activity, little is known of metabolic reactions in the subseafloor crust. Information on possible metabolic reactions is based on analyzed chemical components from rocks and fluids within the crust and vent fluids [19], but also on phylogenetic analysis (i.e. 16s rRNA) from rocks that indicate microbial activity [8, 9, 10]. Alteration of basalt results in acidic fluids enriched in reduced iron, manganese, and sulphur compounds, which are considered, according to thermodynamical modeling, the most likely energy sources for microorganisms in basalt systems [19]. This is supported by molecular analyses and cultivation-based studies performed on seafloor-exposed basalts indicating the presence of microbial groups involved in iron-cycling [5,6,20,21]. With a few exceptions, the community structure in the deep subseafloor basalts and associated microbe-mineral interactions are still unknown [10, 22]. Here we report a fossilized microbial consortium of fungi and prokaryotes, from a depth of 240 m below seafloor (mbsf) at the Detroit Seamount in the Pacific Ocean. We report a complex interplay between the prokaryotes and the eukaryotes that enable microbial growth and colonization.

## Materials and Methods

During ODP Leg 197, the Detroit (Sites 1203 and 1204), Nintoku (Site 1205) and Koko Seamounts (Site 1206) were drilled by the D/V Joides Resolution, see [23] for a map of the sampling area. The samples were not collected in a protected area and specific permissions were not required for sampling. The field studies did not involve endangered or protected species. Of 72 samples examined from all four sites, 17 contained fossilized fungi in open pore spaces [15]. This study is focused on sample 197-1205-34R-5, 33 collected at Nintoku Seamount at a depth of 240.4 mbsf. The drill core sample was investigated under optical light microscopy, environmental scanning electron microscopy (ESEM), and synchrotron-based X-ray tomographic microscopy (SRXTM). Sample 197-1205-34R-5, 33 was cut in smaller pieces to fit in the ESEM or to allow investigation of the interior. Mineral phases were identified and studied by a combination of microscopy, X-ray diffraction (XRD), Mössbauer spectroscopy, infrared spectroscopy, and Raman spectroscopy. The fossils were studied by optical microscopy, ESEM coupled with and energy dispersive spectrometer (EDS), and SRXTM. The investigated materials are deposited in the Palaeobiology collections of the Swedish Museum of Natural History (SMNH X5332–X5340).

For ESEM analyses an XL30 microscope with a field emission gun (XL30 ESEM-FEG) was used equipped with an Oxford x-act energy dispersive spectrometer (EDS), backscatter electron detector (BSE) and a secondary electron detector (SE) (Oxford corp., Oxford, England). The acceleration voltage was normally15 kV but increased to 20 kV if Fe was to be detected in the sample. The instrument was calibrated with a cobalt standard purchased from Oxford instruments. Peak and element analyses were made using the INCA Suite software (version 4.11, Oxford corp., Oxford, England).

X-ray powder diffraction data were acquired using CuK radiation and a Philips PW1050 goniometer (PANalytical B.V., Almelo, The Netherlands) equipped with a graphite monochromator. A background-free Si holder was used for samples that could be extracted only in quantities of <1mg.

Mössbauer spectra were measured with a conventional spectrometer system (Wissenschaftliche Elektronik GmbH, Starnberg, Germany). Because of the small sample size, a ^57^Co Mössbauer point source with a nominal activity of 10 mCi was used. The obtained spectra were calibrated against α-Fe foil and analysed by the least-squares fitting software MDA [24].

Infrared spectroscopy was performed with a Bruker Fourier transform infrared spectroscopy (FTIR) instrument (Equinox 55) (Bruker Optik GmbH, Ettlingen, Germany) equipped with a tungsten source, a CaF_2_ beamsplitter, and an InSb detector. Spectra were measured in the range 2000–5000 cm^-1^ where absorption bands from hydrocarbons are expected to occur. A total of 500 scans were collected in transmission mode for both background and samples, which consisted of unoriented double-polished crystal sections with a thickness of circa 200 μm.

Mineralogical analyses by Raman spectroscopy on untreated pieces of rock samples were performed on two different occasions. The first Raman spectra were recorded at the Department of Earth Sciences, Uppsala University, using a high-throughput, single-stage imaging spectrometer (HoloSpec f/1.8i, Kaiser Optical Systems, Inc., Lyon, France) equipped with a holographic transmission grating and a thermoelectrically cooled (-60°C) CCD detector (Newton, Andor Technology, 1,600 x 400 pixels, Oxford, England), an Ar-ion laser (Spectra-Physics, 514.5 nm, 20 mW, Mölndal, Sweden), and an optical imaging system (magnification 20x, spatial resolution ~1 μm). Two notch filters (Kaiser Optical Systems, Inc., Lyon, France) blocked the Rayleigh line. Raman spectra were collected in backscattering geometry at a resolution and accuracy of about 2 and 1.5 cm^-1^, respectively. Typical acquisition time varied between 1 and 2 min, applying 2–5 mW of the laser.

The second set of Raman spectra was made at the Department of Geological Sciences, Stockholm University, using a confocal laser Raman spectrometer (Horiba instrument LabRAM HR 800; Horiba Jobin Yvon, Villeneuve d’Ascq, France), equipped with a multichannel air-cooled (-70°C) 1024 x 256 pixel CCD (charge-coupled device) detector. Acquisitions were obtained with an 1800 lines/mm grating. Excitation was provided by an Ar-ion laser (λ = 514 nm) source. A low laser power 0.05 mW at the sample surface was used to avoid laser induced degradation of the sample. An Olympus BX41 microscope was coupled to the instrument. The laser beam was focused through an 80x objective with a working distance of 8 mm required to obtain a spot size of about 1 μm. The spectral resolution was ~0.3 cm^-1^per pixel. The accuracy of the instrument was controlled by repeated use of a silicon wafer calibration standard with a characteristic Raman line at 520.7 cm^-1^. The Raman spectra were achieved with LabSpec 5 software (Horiba instrument LabRAM HR 800; Horiba Jobin Yvon, Villeneuve d’Ascq, France).

SRXTM was performed at the X02DA (TOMCAT) beamline [25] at the Swiss Light Source (Paul Scherrer Institute, Switzerland). The examined samples were reduced in size by sawing or breaking and fixed with acetone-soluble glue on aluminium stubs or brass pins. Samples were scanned at beam energies of 18–25 keV depending on sample thickness. The transmitted radiation was converted to visible light using a 20 mm thick LAG:Ce scintillator, magnified with either a 4x or 10x microscope objective and digitized by a sCMOS camera (PCO.edge). The resulting voxel size was 1.625 μm for the 4x and 0.65 μm for the 10x objective. For each scan, 1501 to 2001 projections equiangularly spaced over 180° were acquired, with an exposure time of 125–1250 ms per single projection. Tomographic reconstruction was done using a highly optimized algorithm based on the Fourier method [26], and the obtained tomographic volumes were visualized and rendered using Avizo (FEI corp, Hillsboro, US).

## Results

**Fig 1:**
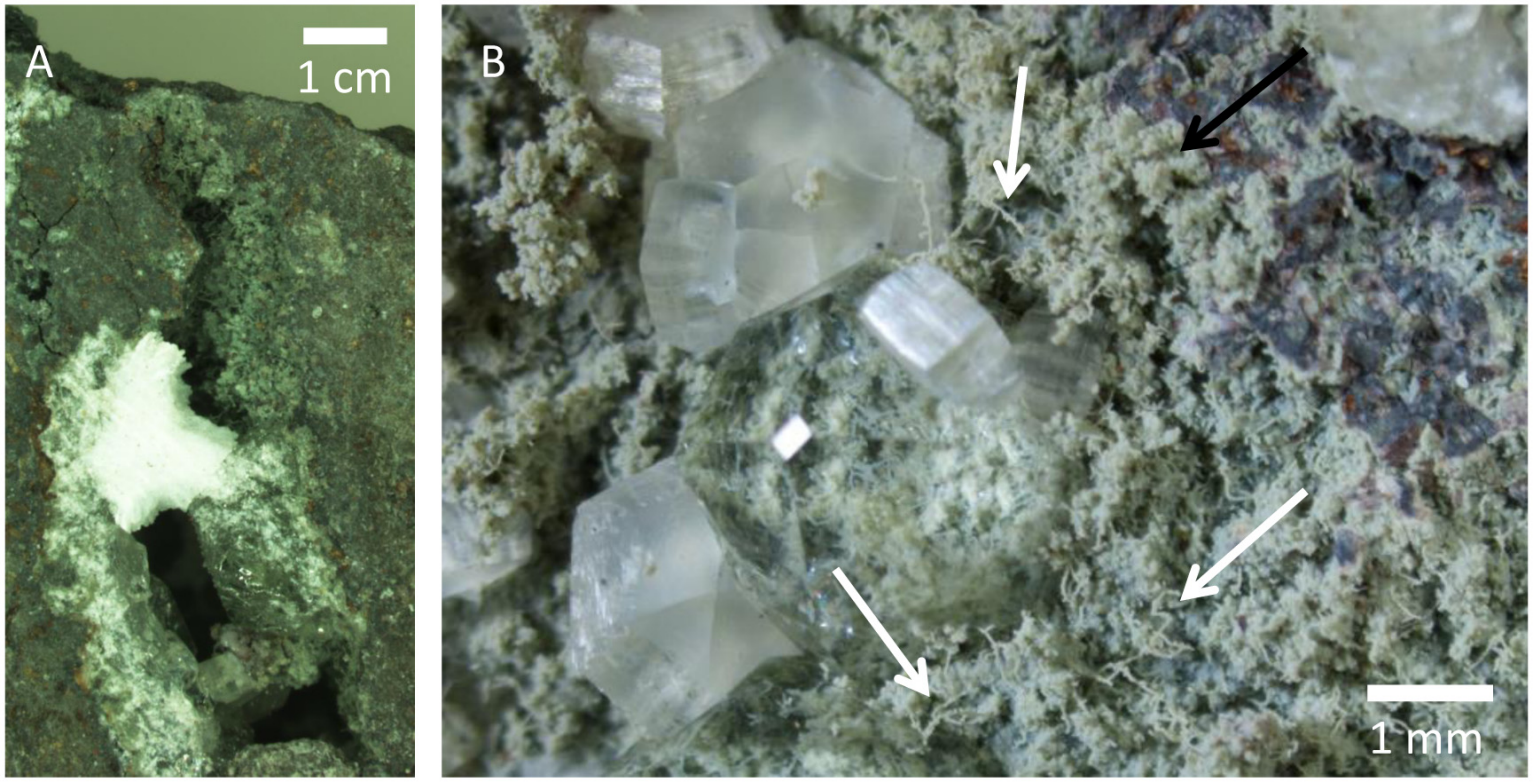
Overview of the vein. Microphotographs of the fracture in sample (SMNH X5332). (A) Overview of the vein. (B) Showing parts of one side of the fracture after splitting. Dense fungal mycelia of yeast-like cells and hypha occasionally overgrown or intergrown by zeolites. Black arrow indicates the fungal mycelia preserved by montmorillonite, white arrows indicates specific hyphae.

Sample 197-1205-34R-5, 33 contains a distinct fracture system that stretches more than 5 cm in length and ~2 cm in width (Fig 1). The fracture is limited by the drill core diameter and represents only a minor part of a larger fracture system. The fracture was mechanically split open to expose the interiors. The walls of the fracture are composed of secondary mineralizations and purported biological structures in an intricate system that makes it difficult to decipher the succession in the system and the internal relationships between the various components.

**Fig 2:**
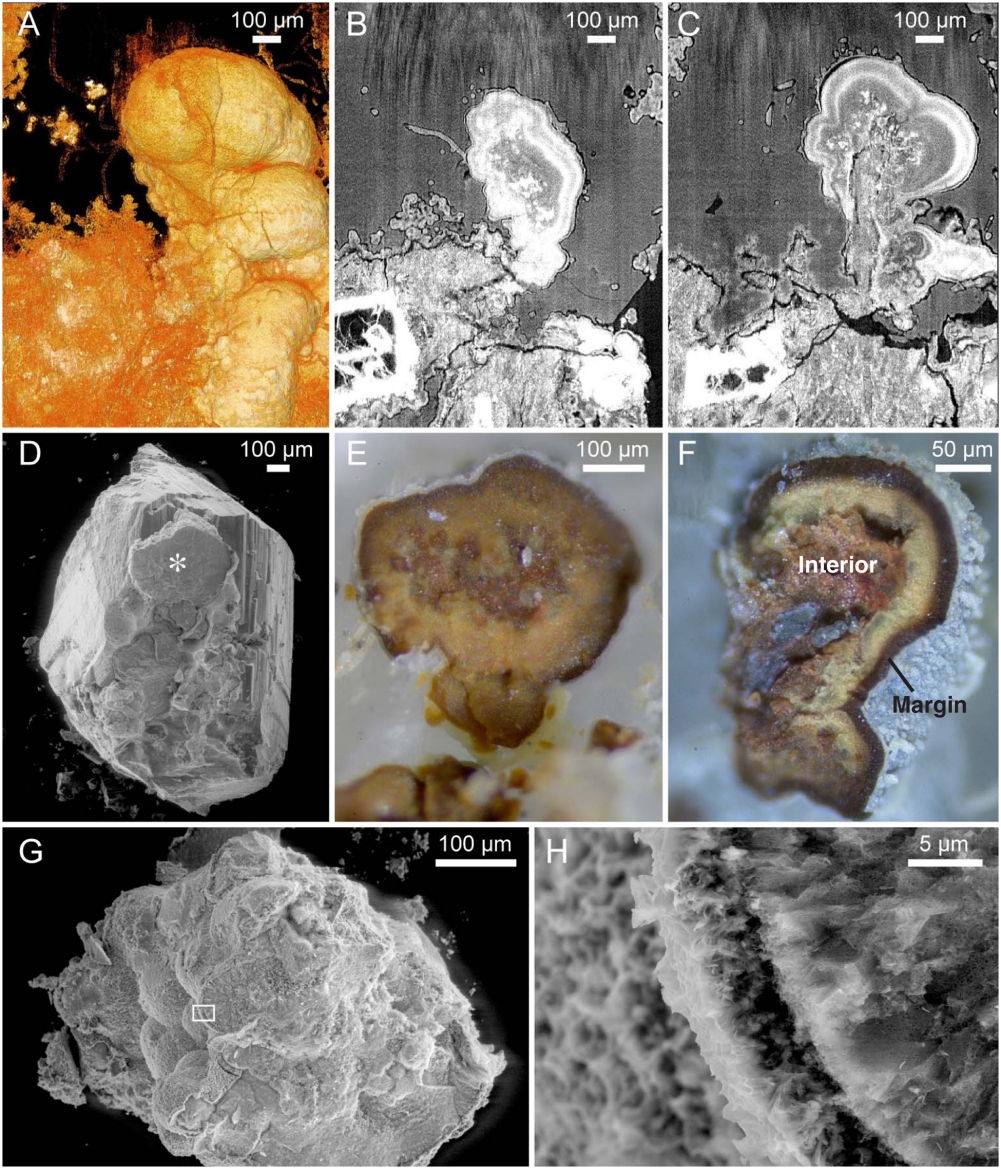
Microstromatolites. (A) Tomographic volume rendering of a multiple microstromatolite at the basalt-vesicle interface (SMNH X5333). (B,C) Tomographic slices of the microstromatolite in A at different levels. The internal structure is characterized by parallel layers in a stromatolitic pattern, and brighter bands towards the margins indicating higher densities. Note how the microstromatolite and surrounding basalt are overgrown by the biofilm and how hyphae protrude. (D) ESEM image of a zeolite crystal (SMNH X5334). A large incorporated microstromatolite is marked with an asterisk. (E) Microphotograph of the marked microstromatolite in D showing a cross section of the internal organization with marginal bands. (F) Microphotograph showing the internal structure of another microstromatolite (SMNH X5335) including layering and the dark marginal band that corresponds to the bright marginal band in SRXTM (compare with B and C). (G) ESEM image of a basalt sample with microstromatolites (SMNH X5336). Frame marks position of H. (H) Close-up of G showing the marginal band.

**Fig 3:**
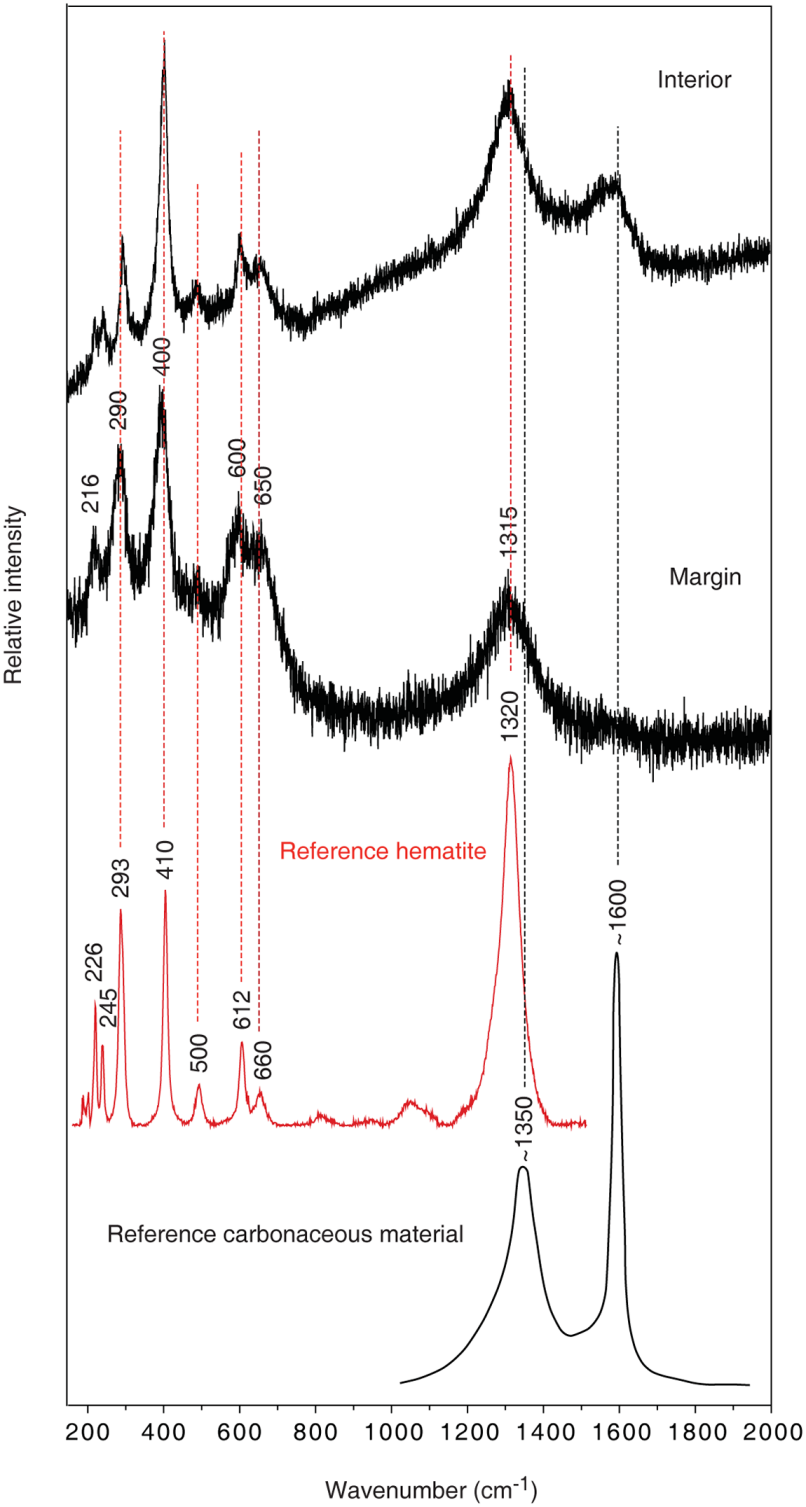
Raman spectra of the microstromatolites. Raman spectra in the spectral range 150–2000 cm^-1^ of the interior and the margin of a microstromatolite (SMNH X5335), shown in 2F. The spectrum from the margin shows bands that are attributed to hematite; a similar spectrum is obtained from the interior but with an additional band around 1600 cm^-1^ that indicates the presence of carbonaceous material. Reference spectra of hematite and carbonaceous material have been incorporated in the figure.

Directly on the basalt are semi-spherical bodies with diameters varying from ~300 μm to ~1 mm, and characterized by relatively smooth surfaces (Fig 2). The structures occur solely or multiply as irregular assemblages, extending from each other in various directions in an almost botryoidal fashion (Fig 2A–2C and 2F). The interiors, exposed by SRXTM, are finely laminated as concentric bands in a “stromatolitic” fashion (Fig 2B and 2C). The marginal bands are brighter in SRXTM, corresponding to higher densities. Specimens with exposed cross-sections also show laminated interior with a distinct dark band at the margins (Fig 2E and 2F) that corresponds to the bright marginal bands in SRXTM. According to Raman analysis they consist predominantly of hematite (Fig 3) [27]. Characteristic for the hematite is that it also shows peaks at 1350 and 1600, indicative of carbonaceous matter (CM) [28]. The most distinct CM peaks are observed in the interiors, and towards the margins CM seems to decline. This is confirmed with EDS, which also shows a declination of C towards the margins. This chemical gradient coincides with the brighter laminations seen in SRXTM, but there are no distinct chemical variations other than decreasing CM. Thus the bands are probably due to more compact crystallization.

**Fig 4:**
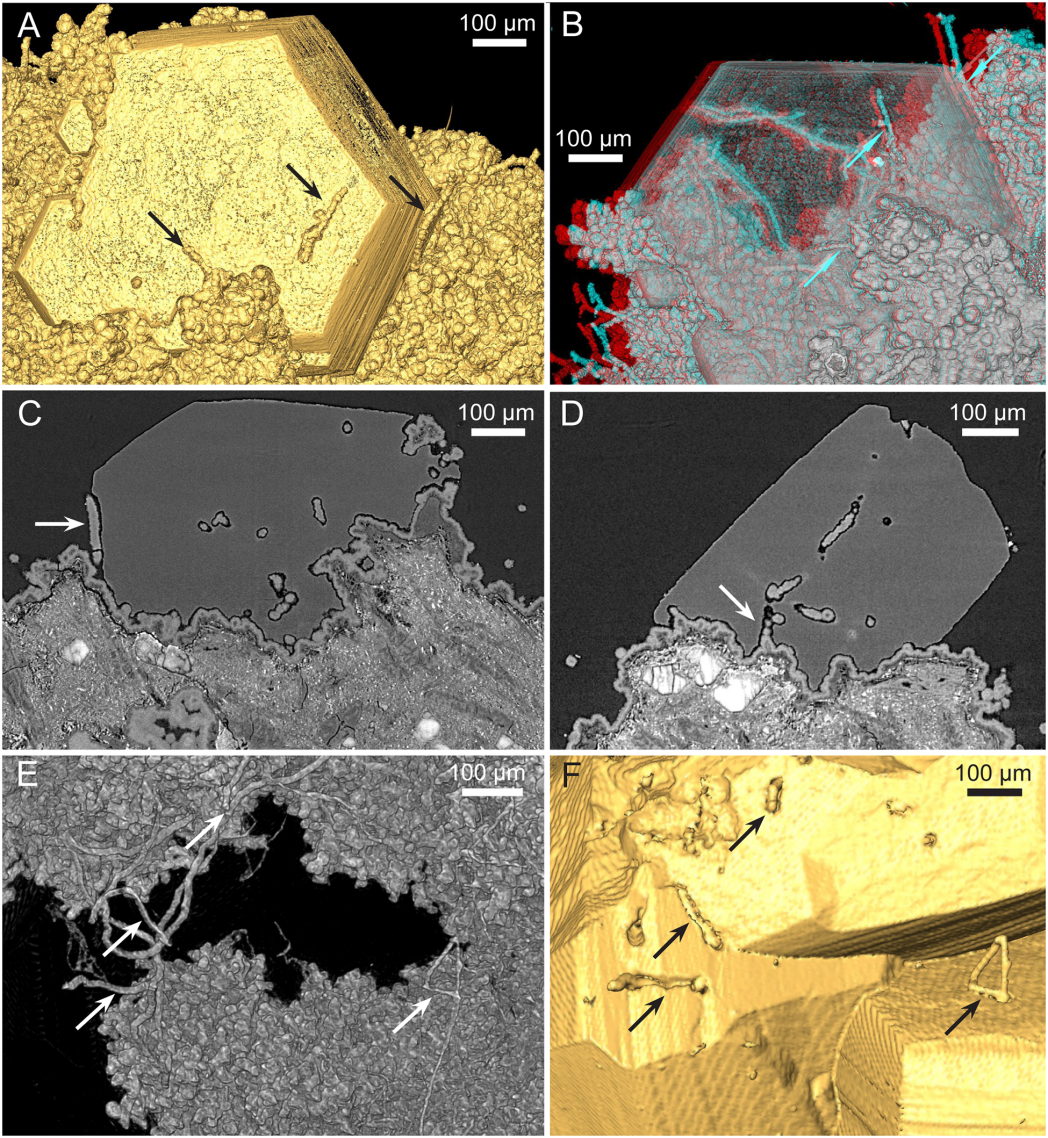
Tomographic renderings of the basalt-clay-zeolite interface showing the biofilm and protruding hyphae. (A–D) SRXTM isosurface (A), volume rendering (B, stereo anaglyph), and slices (C, D) of the cellular biofilm with protruding hyphae (SMNH X5337). The biofilm is partially overgrown by a zeolite crystal. Hyphae creeping along the mineral surfaces (arrows in A–C) leave a negative longitudinal cavity. Arrow in D points to base of the hyphae consisting of repetitive spherical cells that more distally transform into filamentous hyphae. (E,F) SRXTM isosurface (E) and volume (F) rendering showing how hyphae creep along the mineral surface (arrows) and/or branch where they protrude at the surface (right-hand arrows) (SMNH X5333).

The microstromatolitic structures and the basalt in between are covered in an irregular basal film of montmorillonite, ~20 to ~100 μm thick (Fig 1B). The entire fracture walls are lined with this film, resulting in a grey surface highly variable in topography. The film predominantly consists of spherical cell-like structures, ~10 to ~20 μm in diameter (Fig 4A–4E). The cell-like structures occasionally agglomerate into irregular assemblages of ten to hundreds of cells jutting out from the basal film, giving rise to its irregularity.

**Fig 5:**
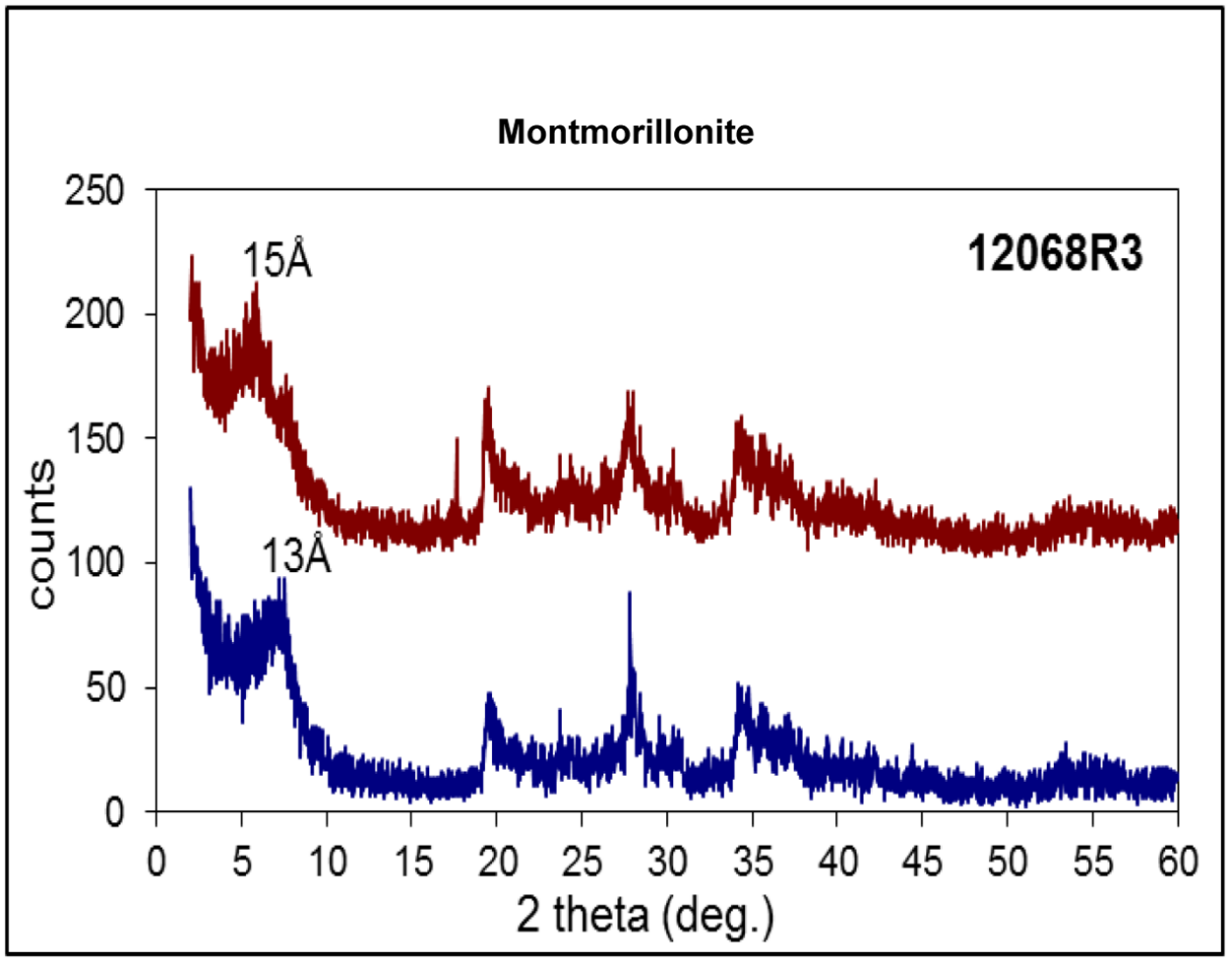
Powder XRD diffractogram of montmorillonite. Note the change of basal reflection from 13 Å for dry sample (bottom) to 15 Å for moist sample (top), indicating basal swelling typical of montmorillonite. A relative low signal/noise ratio due to the small amount of available sample material.

Filamentous hyphae, ~15 to 25 μm in diameter and several hundred micrometres in length, protrude from the basal film forming complex mycelia-like networks in the voids and crystals (Fig 4B–4E). The base of the hyphae consists of repetitive spherical cells from the basal film that subsequently transform into filamentous hyphae (Fig 4D, arrow). The hyphae branch frequently but also form short characteristic stub-like branches, ~5 to 10 μm in length. Both hyphae and the cell-like structures are mineralized by montmorillonite, just as the basal film (Fig 5).

**Fig 6:**
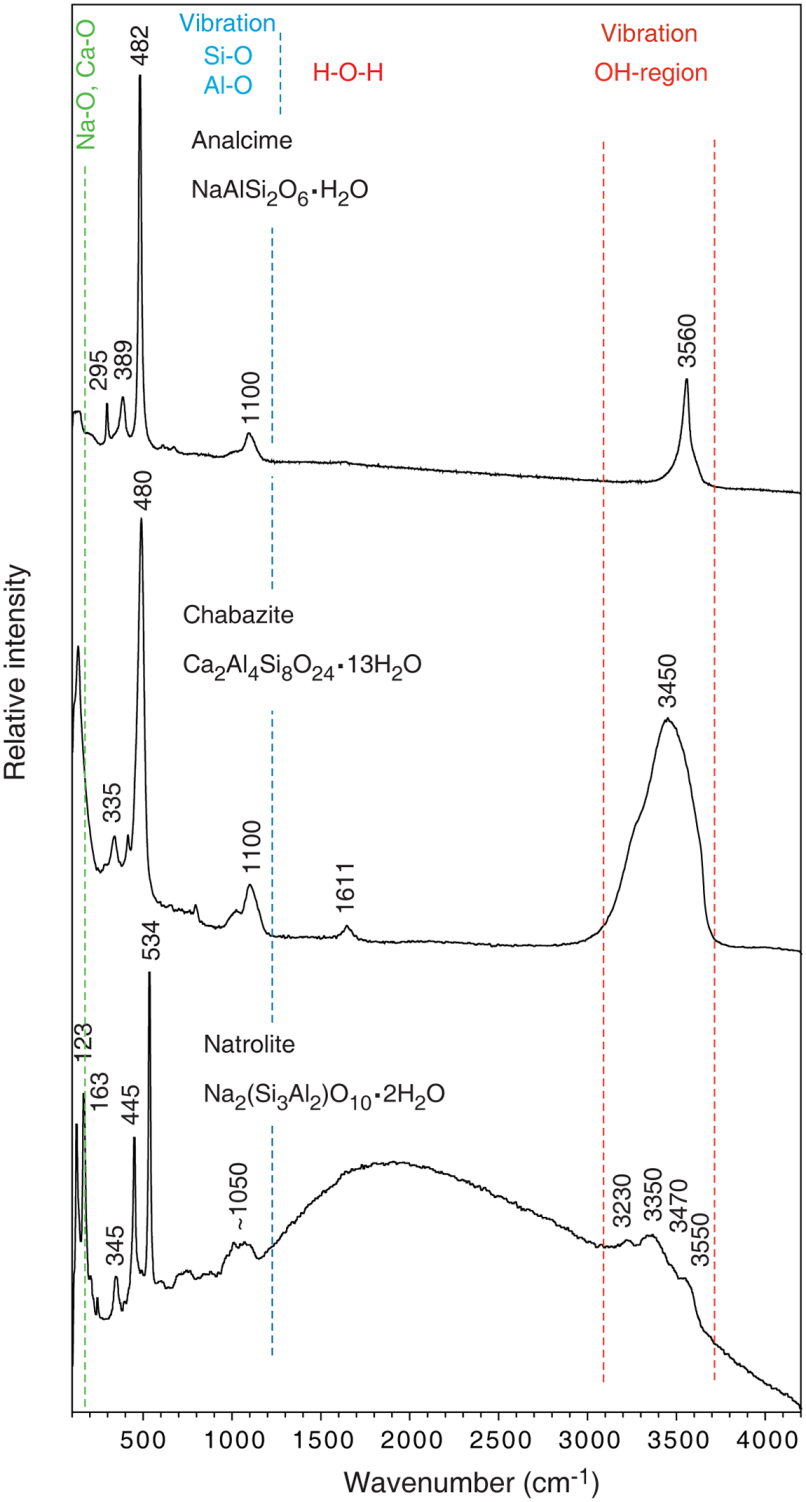
Raman spectra of zeolites. Raman spectra in the spectral range 100–4200 cm^-1^ of the zeolites that are identified as analcime, chabazite and natrolite after comparison with reference spectra in [27]. An idealized chemical composition is given for each zeolite mineral. Simplified characteristic wavenumber ranges for different Raman vibrational modes of the zeolite spectra are marked with different colours where green (<200 cm^-1^) is assigned to Ca-O and Na-O, blue (300–1200 cm^-1^) to Si-O and Al-O and red (1611 cm^-1^ and 3100–3700 cm^-1^) to O-H vibrations.

**Fig 7:**
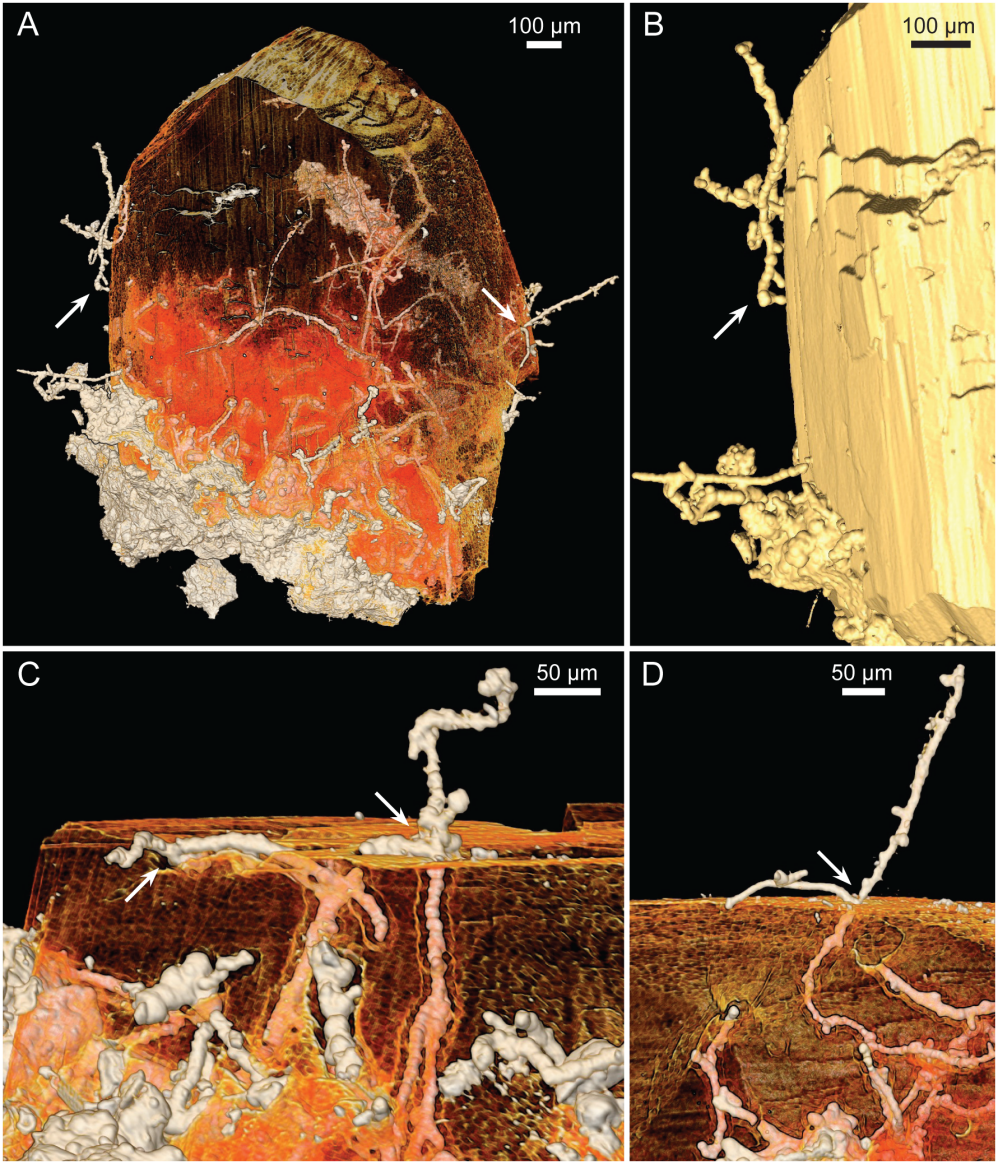
Tomographic renderings of boring hyphae protruding on the zeolite surface (SMNH X5338). (A) Volume rendering showing abundance of hyphae within a zeolite crystal and how they protrude at the mineral surface. Arrow at the left shows hyphae taking a path above the mineral surface; arrow to the right shows hyphae that branch at the point where they exit the mineral surface. (B) Isosurface rendering of left side of A showing a hypha taking a path above the mineral surface, occasionally touching it with short branches. (C, D) Volume rendering showing protruding hyphae that branch at the point where they exit the mineral surface, one branch creeping along the surface.

The distinct fracture within a particular Nintoku Seamount sample is characterized by abundant zeolite growth. The basal film is occasionally overgrown by zeolites (chabazite, analcime and natrolite) that occur as millimetre-sized crystals (Figs 1, 4 and 6). Chabazite and analcime occur as euhedral crystals, transparent or semi-transparent. Natrolite is fibrous and white opaque. The basal film with hyphae and cell-like structures within the zeolites is similar in appearance to that outside. Both hyphae and agglomerations of cell-like structures frequently protrude hundreds of micrometres from the basal film (Figs 4 and 7). Commonly they stretch from the basal film, throughout the zeolite crystal and reach the surface where they protrude either into a different zeolite or into the fracture void. Some hyphae protrude from one zeolite surface into the void and penetrate an opposite zeolite surface. However, the film never covers the zeolites.

**Fig 8:**
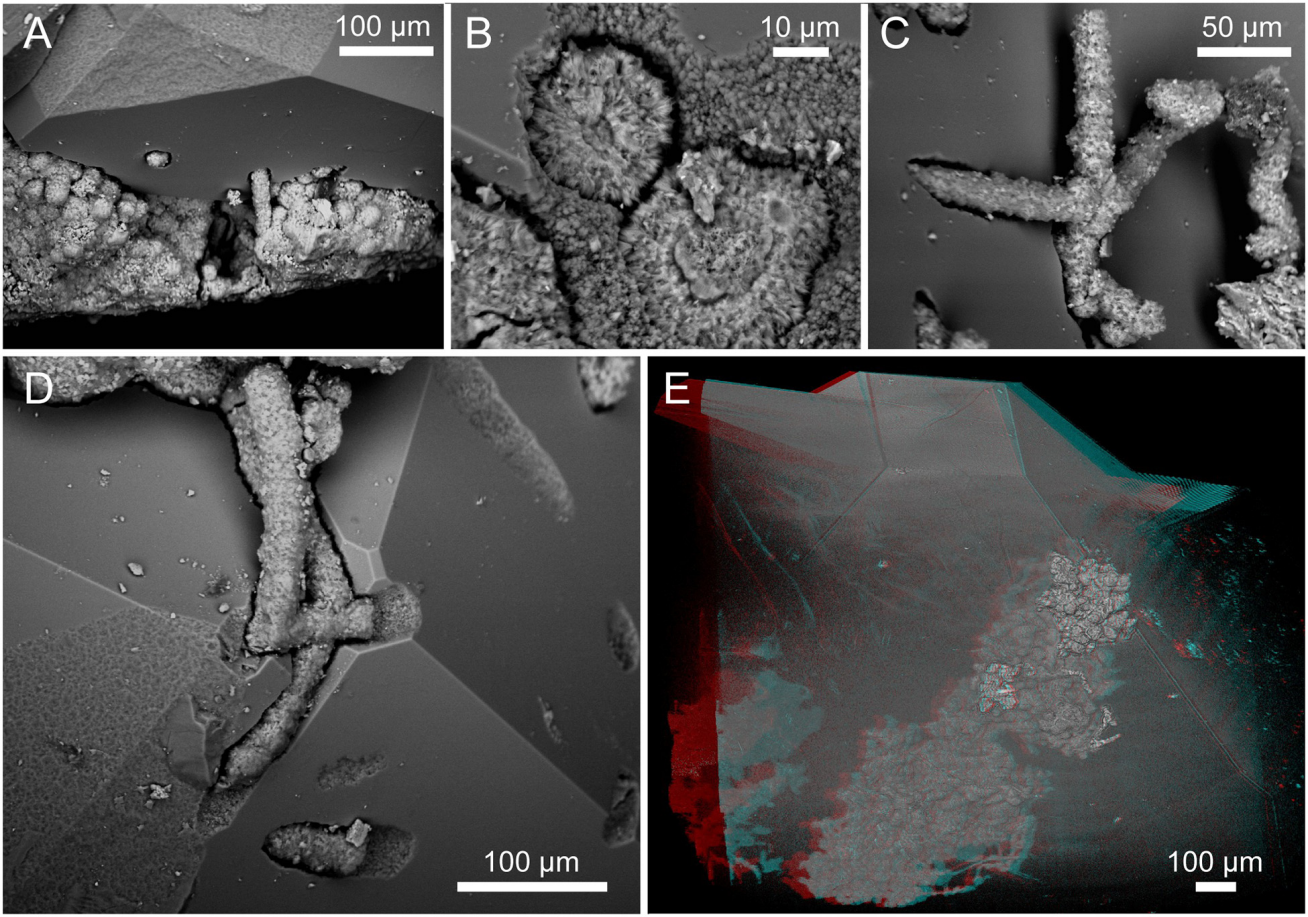
ESEM (A–D) (SMNH X5339) and SRXTM (E) (SMNH X5340) images of fungi influencing the mineral surface. (A) The influence of both yeast and hyphae on the mineral surface leaving negative pits. (B) The contact between two protruding hyphae and the zeolite surface. Note the rough and irregular texture of the mineral surface at contact compared to the normally smooth surfaces. (C) Hyphae protruding angularly and creeping along the mineral surface. (D) Hyphae creep along the mineral surface and their influence on the zeolite surface at contact. (E) Stereo anaglyph of an assemblage of cells within a zeolite crystal.

Both hyphae and cell-like structures occur inside the zeolites in cavities corresponding in size and morphology to the fossilized organisms; hyphae in tunnels, and cell-like structures as negative spherical pits (Figs 4A–4D, 7C and 8A–8D). At the zeolite surfaces the behaviour of the hyphae differs. Some hyphae just protrude perpendicularly or angularly but on a straight path from the inside of the zeolites, apparently unaffected by the change of medium. However, in many instances hyphae seem to react to the presence of the mineral surface, by following the surface (Fig 4A–4C, 4E and 4F, arrows, Figs 7, 8C and 8D), by taking a path above the surface, occasionally touching it with short branches (Fig 7A, left arrow, Fig 7B, arrow), or by branching at the point where they exit/enter the mineral (Fig 7A, right arrow, Fig 7C and 7D, arrows). Where hyphae are in direct contact with the zeolites the zeolite surfaces are rough and irregular and deviate from the otherwise smooth and well defined crystal surfaces (Fig 8B and 8D).

**Fig 9:**
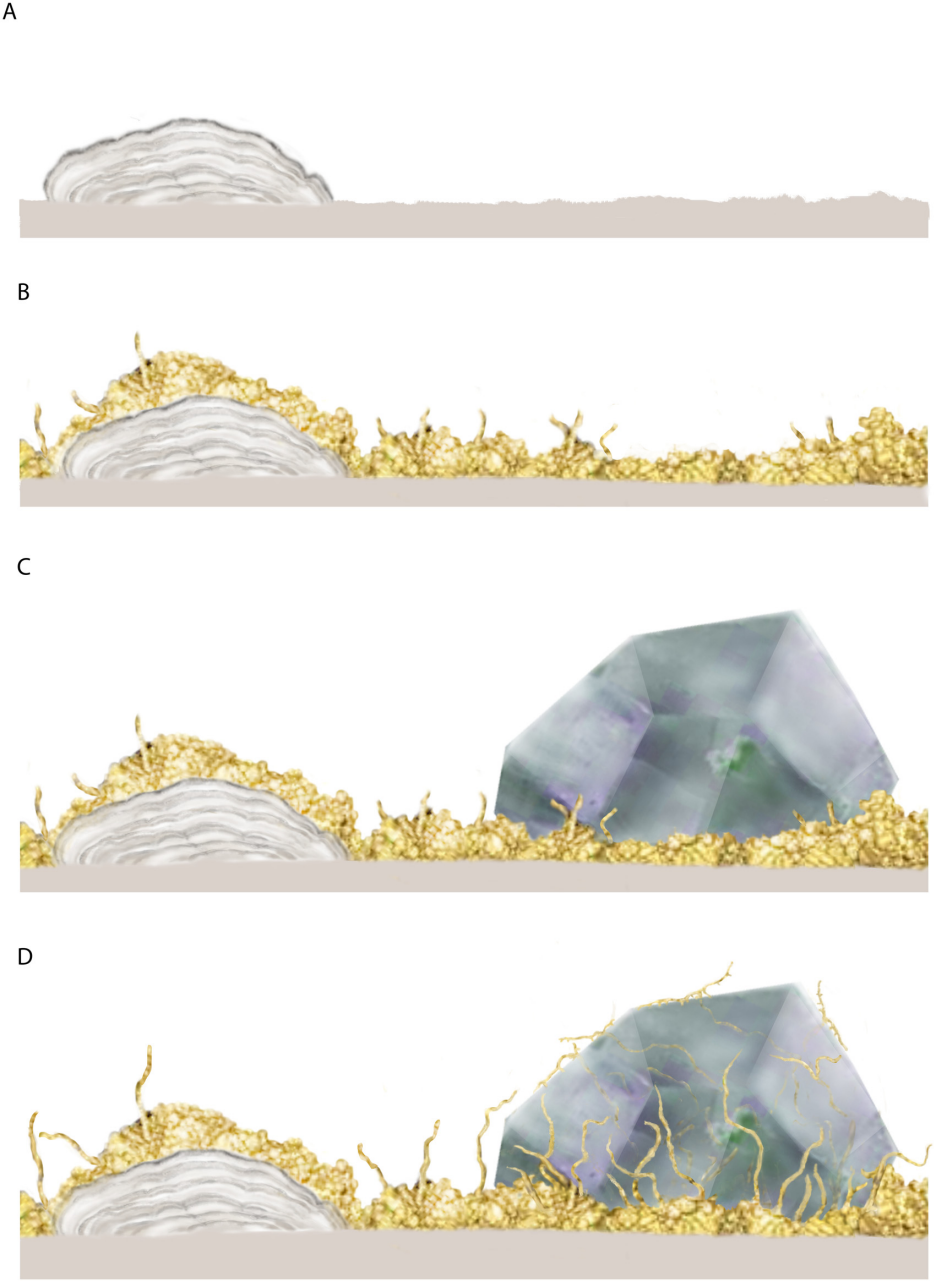
Illustration of the successive colonization and mineralization in the fracture system. (A) Colonization of the microstromatolites on the basalt. (B) Colonization of the fungal biofilm with yeast cells and hyphae, and subsequent overgrowth of the microstromatolites. (C) Partial zeolite overgrowth of the fungal community. (D) Hyphal growth outside and inside of the zeolite crystals. Fungal hyphae bore tunnel-like structures through the zeolite until they reach the mineral surface. At the zeolite surface, hyphae branch, creeps along the surface, occasionally touching it with short branches.

To better constrain the succession in the sample Fig 9 shows the stepwise colonization and mineralization in the fracture system.

**Fig 10:**
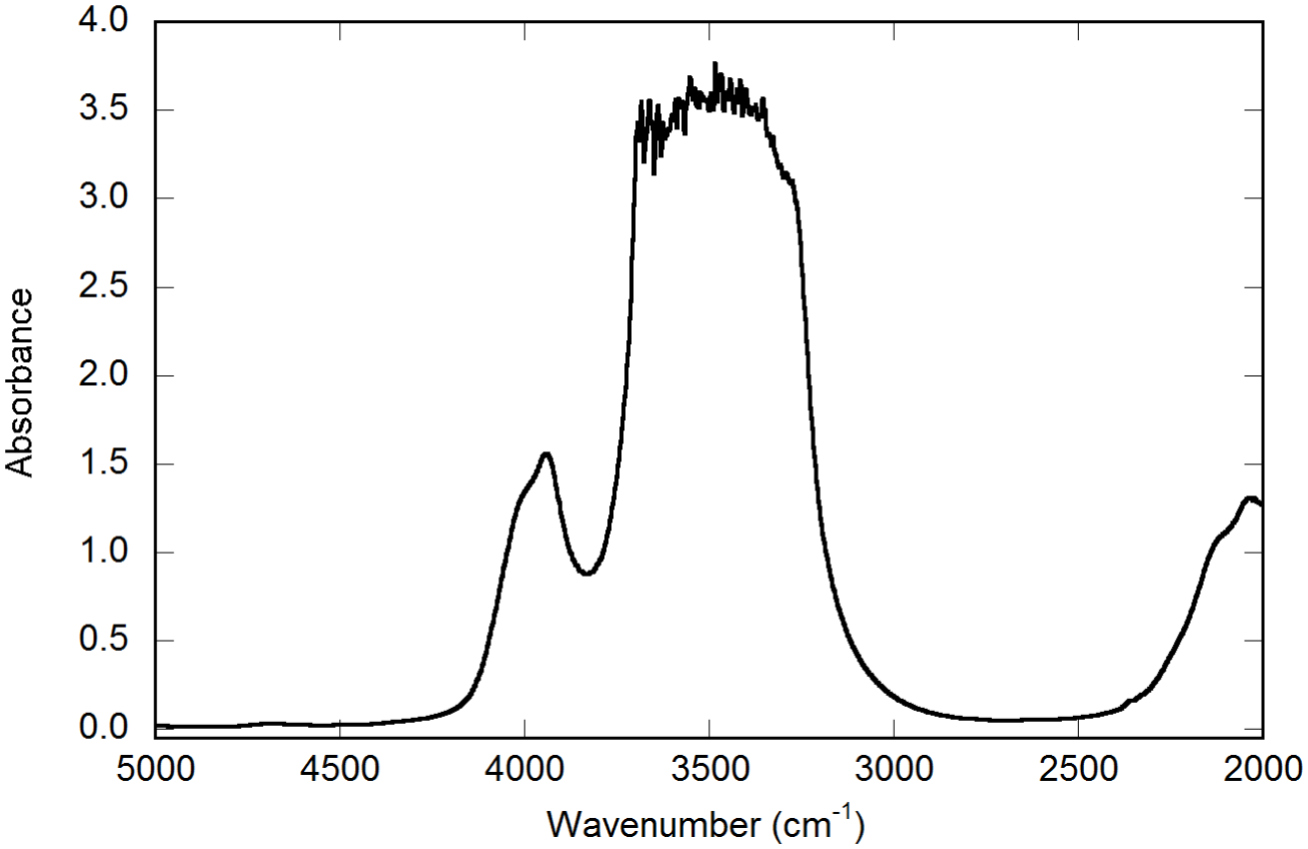
IR spectra of chabazite. (A) FTIR spectrum of chabazite single crystal showing bands caused by the chabazite structure. Sample thickness is ca 200 μm. The main band centred at 3500 cm^-1^ is caused by water molecules (crystal water), whereas the bands around 2100 and 4000 cm^-1^ are caused by crystal lattice overtones. Raman spectrum of the same chabazite crystal in Fig 6 confirms the absence of hydrocarbons and the presence of molecular water.

**Fig 11:**
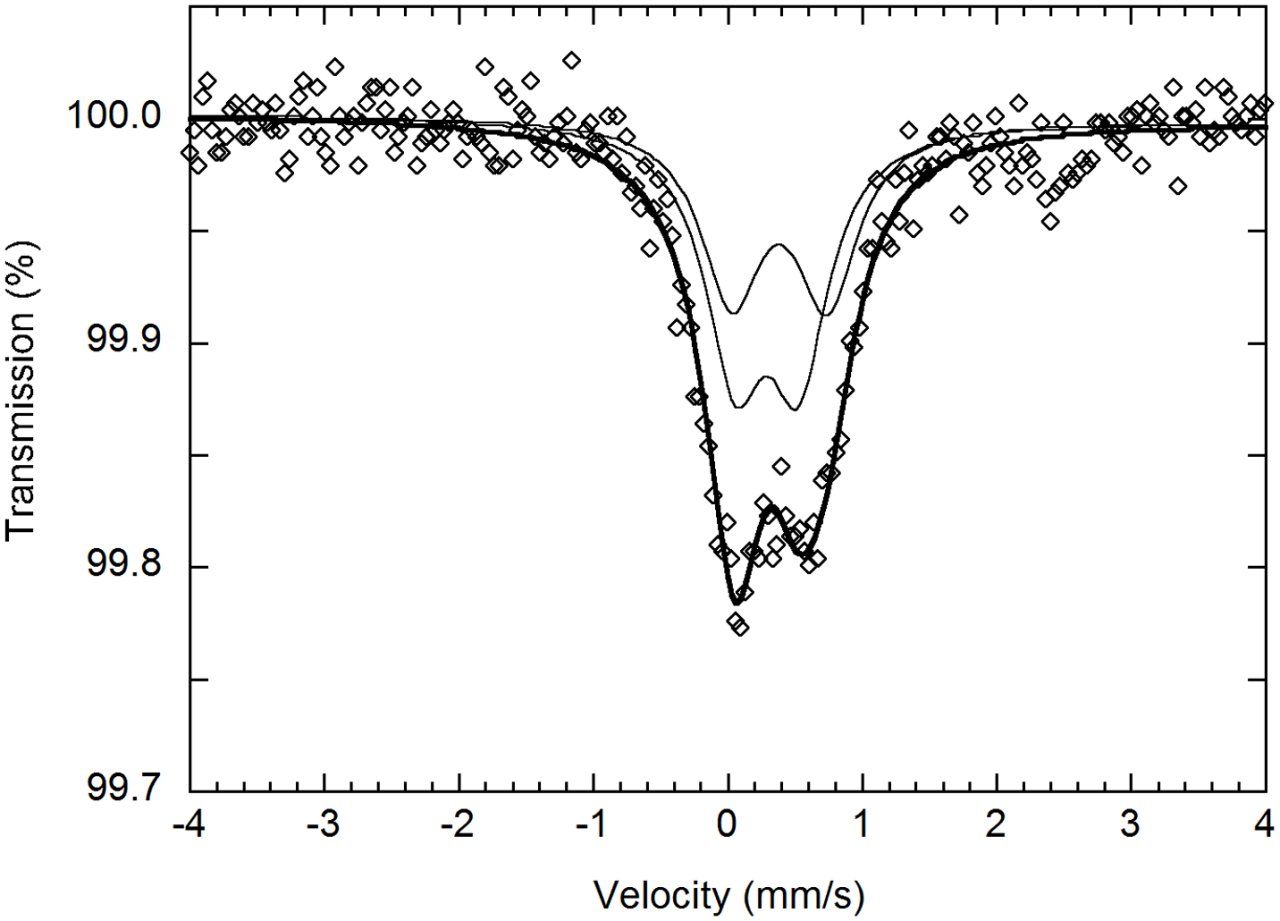
Mössbauer spectrum. Mössbauer spectrum of filamentous structures embedded in a zeolite crystal. Diamonds represent measured spectrum, thick solid line represents the sum of the two fitted doublets (thin lines) assigned to Fe^3+^. The obtained hyperfine parameters are similar to those reported for Fe-bearing montmorillonite [29].

Zeolites contain cavities or tunnels and were analysed by both IR and Raman spectroscopy to detect mineral inclusions or gas or liquid hydrocarbon compounds adsorbed within or on the zeolite crystal framework. IR spectroscopy and Raman spectroscopy detected only crystal water in the zeolites (Figs 6 and 10). Mössbauer analysis of the Fe content of the hyphae in zeolites showed that Fe occurs exclusively as Fe^3+^, with hyperfine parameters closely resembling those reported for Fe-bearing montmorillonite [29] (Fig 11).

## Discussion

### Microstromatolites

Ferruginous microstromatolites have previously been reported from marine and hydrothermal environments, and are mostly interpreted as the remains of microbial activity, usually iron oxidizing *Bacteria* but also fungi [30, 31]. Biogenicity of stromatolitic structures is subject to some uncertainty and there are discussions on how to distinguish between biogenic stromatolites and non-biogenic precipitates [32]. We interpret the Nintoku Seamount microstromatolites to be microbial in origin, for three main reasons:

1. The occurrence and morphology corresponds to that of ferruginous microstromatolites previously reported from marine environments and interpreted as bacterial remains of iron oxidizing *Bacteria* [31]. The multiple microstromatolites resemble bacterial shrubs from hydrothermal environments also interpreted as bacterial remains of iron oxidizing *Bacteria* [33]. Such bush-like structures represent the biological end of a continuum from bacterially mediated “shrubs” to abiotically precipitated dendrites dominated by distinct crystal shapes. Bacterial iron oxidation results in the production of Fe^3+^ species like Fe-oxy/hydroxides such as goethite, or Fe^2+^/Fe^3+^ species like hematite [30,33].
2. The high content of CM indicates the presence of decomposed organic remains in the structures. Organic compounds occur in fluids at seafloor vents and their origin can be biological or abiotic. Considering the overall biological interpretation of these microstromatolites and that the CM is only located at the microstromatolites and absent in other mineral phases with affinity for organic compounds, like the montmorillonite, we interpret the CM as remains of the original bacterial community.

CM is closely associated with, or even incorporated in, the hematite of the microstromatolites. Fe and organic C are biogeochemically strongly interlinked, Fe is known to stabilize and preserve organic C, but organic C also complex and stabilize Fe [34]. The pathways for microbial Fe stabilization are not fully understood, but organic ligands are known to stabilize Fe, and cellular uptake of Fe can be preserved for long periods of time upon cell death [35].

The Nintoku Seamount microstromatolites represent remains of bacterial communities involved in Fe oxidation. Basalts contain substantial amounts of reduced Fe, and the stromatolite-building microorganisms probably mediated oxidation of the Fe in the basalts, which was complexed by and incorporated in the microbes and associated biofilm. Subsequent death of the microbes and formation of new layers of biofilm incorporated the organically complexed Fe in the overall stromatolitic structure. Subsequently the microbial community mineralized to hematite that stabilized the high content of organic matter in the structure.

### Fungi

The biogenicity of the described fungal communities has already been discussed and established by Ivarsson et al. [15]. The present study further characterizes the fungal community, and extends the understanding of fungal-prokaryotic deep-biosphere consortia and the microbial interaction with secondary mineral growth.

The hyphae and cell-like structures originate from, or represent an integral part of, the basal film. Thus, the basal film is the first step in the fungal colonization. Compared to Bengtson et al. [18], who described from the Koko site a basal fungal film organized in distinct layers of hematite/carbon, hematite, and montmorillonite, the current basal film is predominantly made up of the cell-like structures. In a fungal context these could be referred to as yeast-like growth structures. Another difference from the Koko subseafloor fungal hyphae is that the Nintoku hyphae appear to originate from repetitively organized cells, forming smooth transitions between the two growth stages. Fungi exposed to environmental stress sometimes develop monilioid hyphae—specialized hyphae composed of compact cells [36]. Subseafloor basalts are certainly stressful settings for microorganisms with respect to nutrient availability, fluctuating temperatures, salinity and geochemistry, thus, such an organismal organisation is plausible.

Bengtson et al. [18] reported a symbiosis-like relationship between fungi and prokaryotes, an organization that facilitated the eukaryotic colonization and persistence in deep subseafloor basalt. Chemoautotrophs, suggested to be involved in iron oxidation, fixed C from fluids and made it available for the heterotrophic fungal growth. In the Nintoku Seamount samples the microstromatolites are overgrown, and most likely scavenged, by the fungi. The lack of CM in the stromatolitic margins may be a result of fungal scavenging of CM. Beside the obvious access to metabolically important carbohydrates, the prokaryotic community could also provide Fe(II) complexed in the organic matter [35]. Fe(II) is important in metabolic and cellular processes, and the fungi would gain both carbohydrates and bioavailable Fe(II) by grazing the prokaryotic stromatolitic communities. Under this interpretation, chemoautotrophic iron oxidation by the *Bacteria* represents the base of the microbial community in the Nintoku Seamount samples and enables heterotrophic growth and colonization of the fungal community portion.

### Fungi-zeolite interface

The intricate relationship between the hyphae and the zeolite surfaces indicates that the formation of zeolites and the growth of the fungal communities were contemporaneous and that the fungi were alive during zeolite formation, rather than being enclosed by zeolites after fossilization. Hyphae creeping along mineral surfaces show that the growth direction in some cases was controlled by already existing mineral surfaces. Change of growth direction and branching at the mineral surface further suggest that the hyphal growth was influenced by the presence of the mineral surface, indicating active boring by the hyphae. These observations argue against the possibility that the fungi in all cases were encapsulated by the zeolite formation.

A similar scenario but including carbonates instead of zeolites was described by Bengtson et al. [18], where a fungal biofilm remained intact during calcite overgrowth and produced hyphae that actively bored in the calcite after its formation. Hyphae protruding from non-covered parts of the basal films used crevices in the calcite for migration and penetration, showing that the fungal community actively bored in the calcite after its formation. Zeolites do not have surface crevices as scalenohedral calcite has, but the report of Bengtson et al. [18] provides a model for concurrent microbial/mineral growth, and we argue for a similar microbe-mineral succession in the current samples with the difference that there is no direct hyphal penetration at the bare mineral surfaces in the present material. Hyphae creeping along the surfaces have produced negative channels or pits, most likely by dissolution, but no deep galleries start from the mineral surfaces. Instead, all extensive fungal networks start at the basal film and propagate towards the mineral surface. This does not exclude the possibility that some hyphae formed prior to the mineral formation and were encapsulated, but hyphae that clearly interact with the mineral surfaces as described are interpreted as active borers.

The hyphal propagation within the zeolites appears not to be controlled by the crystal lattice, thus it is probably the result of chemical dissolution rather than physical force [37, 38]. Microorganisms have previously been found to dissolve and produce etch-pits in subseafloor zeolites [39, 40], but microbial weathering of this magnitude involving complex tunnel structures has not been described before. Fungi are known from other environments, like soils, to weather a wide range of minerals chemically including carbonates and silicates by producing organic acids and siderophores [37, 38]. However, their involvement in the production of tunnels in minerals and substrates has been widely discussed and is still unresolved [41], mainly because the responsible microorganisms are never found in the tunnels. This is in contrast to our findings, where the organisms remain and have been preserved in the produced structures.

Acquiring habitable space within a hard substrate, such as a mineral, bone or shell, is an adaptive strategy that has evolved in several lineages, including *Bacteria*, fungi, algae and phyla of the Metazoa [42]. Many microborers are photosynthetic, including cyanobacteria and red and green algae, and the advantages with boring into a substrate are usually not trophic but the production of residential cavities [42]. However, in a nutrient-poor environment such as subseafloor basalts, trophic reasons should be considered. Extensive mineral tunnelling by fungi has been reported to be more intense in nutrient-poor sites [37]. It has been suggested that microtubular ichnofossils in subseafloor volcanic glass are produced by chemolithoautotrophic microorganisms that oxidise Fe(II) or Mn(II) in the glass [43]. Zeolites do not normally contain any element or compound with redox potential available for microorganisms. Zeolites are, on the other hand, well known for their capacity to absorb various elements and compounds like metals, hydrocarbons and molecular hydrogen within their crystal framework [44, 45]. All those compounds are common in hydrothermal environments, and microorganisms could get access to them when dissolving the minerals. Neither IR nor Raman analysis of the zeolites revealed any compounds bound within the zeolite framework. However, metals like Ca, Na and K are essential for fungal growth and metabolism [37, 38] and easily accessible in the zeolites. Chabazite is a Ca-, Na-, K-zeolite species; and analcime and natrolite are Na-zeolites. Furthermore, the development of microbial consortia coincides with the formation of secondary minerals [18]. The microbial growth might have been triggered by the instant access to essential metals.

We also need to consider the spatial relationship between the fungi and the zeolites. Normally, in the context of microbial mineral etching the responsible microorganism attacks the substrate at a bare surface. In the current situation the microorganisms have been overgrown by minerals and thereafter produced tunnel structures stretching from the bottom to the top, thus the fungal boring might be a response to environmental stress.

According to the core logging in the ODP Initial Reports vesicles and veins in sample 197-1205-34R-5, 33 are “partly filled with carbonate, dark greenish gray clay, or Fe oxyhydroxides” [46]. These observations are correct, but our results add another dimension and show that some of these seemingly abiotic mineral phases including the greenish gray clay and the Fe oxyhydroxides are biological in origin. The same goes for many more of the samples collected during ODP Leg 197 [15, 18]. We encourage a change of view regarding deep basalt samples that involves a more detailed investigation of new samples but also re-evaluation of old ones. The seemingly abiotic mineral predominance in basalt voids sometimes holds information on a biological microcosm just waiting to be unravelled.

## Conclusions

The basalt-clay-zeolite interface in a sample from 240 m depth below seafloor collected at the Nintoku Seamount in the Pacific Ocean was investigated with respect to purported biological structures. A fossilized microbial community of both prokaryotes and fungi was revealed with an intricate relationship to its physical surrounding. The base of the microbial community is represented by microstromatolites—structures formed by iron oxidizing *Bacteria* that were involved in the oxidation of iron from the basalt. The microstromatolites are overgrown by a fungal community consisting of a basal film, yeast-like growth structures, and hyphae that scavenged the prokaryotic portion of the microbial consortia and, thus, enabled a heterotrophic, eukaryotic colonisation and persistence in the deep basalts. The fungi are in turn overgrown by secondary zeolites but stayed active during this course of event. After the zeolites were formed they were actively bored by the fungi producing tunnel-like structures in the mineral substrate. The fungal boring in the zeolites could either be for trophic reasons gaining metals like Ca, Na, and K, essential for fungal growth, or a response to the environmental stress that the zeolite overgrowth induced upon the fungi. In summary, the mineral succession at the basalt interface in the Nintoku Seamount sample reveals an intricate microbial community structure and its interplay with its physical surrounding that increases our understanding of the deep ecology, and indicates that paleontological material is an inexhaustible source of information in the exploration of the deep biosphere of subseafloor igneous crust.
